# Cholesterol uptake and efflux are impaired in human trophoblast cells from pregnancies with maternal supraphysiological hypercholesterolemia

**DOI:** 10.1038/s41598-020-61629-4

**Published:** 2020-03-24

**Authors:** Bárbara Fuenzalida, Claudette Cantin, Sampada Kallol, Lorena Carvajal, Valentina Pastén, Susana Contreras-Duarte, Christiane Albrecht, Jaime Gutierrez, Andrea Leiva

**Affiliations:** 1 Division of Obstetrics and Gynaecology, School of Medicine, Faculty of Medicine, Pontificia Universidad Católica de Chile, Santiago, Chile; 2 Institute of Biochemistry and Molecular Medicine, Faculty of Medicine, University of Bern, Bern, Switzerland; 3 Swiss National Centre of Competence in Research, NCCR TransCure, University of Bern, Bern, Switzerland; 4 School of Medical Technology, Health Sciences Faculty, Universidad San Sebastian, Santiago, Chile

**Keywords:** Dyslipidaemias, Endocrine reproductive disorders, Endocrine reproductive disorders

## Abstract

Maternal physiological (MPH) or supraphysiological hypercholesterolaemia (MSPH) occurs during pregnancy. Cholesterol trafficking from maternal to foetal circulation requires the uptake of maternal LDL and HDL by syncytiotrophoblast and cholesterol efflux from this multinucleated tissue to ApoA-I and HDL. We aimed to determine the effects of MSPH on placental cholesterol trafficking. Placental tissue and primary human trophoblast (PHT) were isolated from pregnant women with total cholesterol <280 md/dL (MPH, n = 27) or ≥280 md/dL (MSPH, n = 28). The lipid profile in umbilical cord blood from MPH and MSPH neonates was similar. The abundance of LDL receptor (LDLR) and HDL receptor (SR-BI) was comparable between MSPH and MPH placentas. However, LDLR was localized mainly in the syncytiotrophoblast surface and was associated with reduced placental levels of its ligand ApoB. In PHT from MSPH, the uptake of LDL and HDL was lower compared to MPH, without changes in LDLR and reduced levels of SR-BI. Regarding cholesterol efflux, in MSPH placentas, the abundance of cholesterol transporter ABCA1 was increased, while ABCG1 and SR-BI were reduced. In PHT from MSPH, the cholesterol efflux to ApoA-I was increased and to HDL was reduced, along with reduced levels of ABCG1, compared to MPH. Inhibition of SR-BI did not change cholesterol efflux in PHT. The TC content in PHT was comparable in MPH and MSPH cells. However, free cholesterol was increased in MSPH cells. We conclude that MSPH alters the trafficking and content of cholesterol in placental trophoblasts, which could be associated with changes in the placenta-mediated maternal-to-foetal cholesterol trafficking.

## Introduction

During the progress of human pregnancy, the concentrations of total cholesterol (TC) and low- (LDL) rises in a physiological (maternal physiological hypercholesterolemia, MPH)^1,2^ or supraphysiological (maternal supraphysiological hypercholesterolemia, MSPH)^3,4^ way. MSPH is determined in women with TC levels above a cut-off value of 280–300 mg/dL at term of pregnancy or above the 75th percentile for the different trimesters of pregnancy^1–6^.

Endothelial dysfunction of the macro- and the microvascular vessels of the placenta^4,7–9^ as well as development of atherosclerosis in the foetal aorta^1,2,6^ has been described in pregnancies with MSPH and increased LDL levels. This information suggest that MSPH could be related to the development of cardiovascular disease in the offspring later in life^2,6,10,11^.

Despite the increased maternal TC and LDL concentrations, the levels of TC and triglycerides (Tg) of neonates from MSPH pregnancies are comparable to those from MPH pregnancies, suggesting a possible regulation of placental maternal-to-foetal cholesterol trafficking across the placenta^7,12^.

The syncytiotrophoblast is a multinucleated and polarized tissue originated by fusion of cytotrophoblast cells. The syncytiotrophoblast is responsible for the maternal-to-foetal exchange of nutrients. This tissue present an apical side in contact with the maternal blood and a basal membrane in contact with the foetal endothelial cells. Therefore, the expression of receptor or transporter proteins on one or the other side of the syncytiotrophoblast membrane determines the directionality of the released molecules.

Maternal-to-foetal cholesterol trafficking requires that syncytiotrophoblast take up cholesterol from maternal LDL and HDL through the endocytic LDL receptor (LDLR) and the HDL receptor scavenger receptor class B type I (SR-BI), respectively. Then, the cholesterol is released through cholesterol transporters such as ATP-binding cassette transporter A1 (ABCA1) and ABCG1 to the placental endothelial cells and subsequently to the foetal circulation^13–15^. Although most of the events related to these phenomena remain unclear, the principal proteins involved in maternal cholesterol uptake and efflux to the foetal circulation have been identified in the human placenta^13,14^. In the placenta, endothelial cells and the syncytiotrophoblast express LDLR^16,17^, SR-BI^18,19^, ABCA1 and ABCG1, which mediate the uptake of cholesterol and its release from cells to acceptors such as ApoA-I (via ABCA1) or HDL (via ABCG1)^19,20^.

Interestingly, in cells such as macrophages, SR-BI participates not only in the uptake of HDL but also in the efflux of cholesterol to acceptors such as ApoA-I and HDL^21,22^. Despite that, in endothelial cells from the placenta, SR-BI does not contribute to this process^19^. However, the possible contribution of this receptor in the efflux of cholesterol in trophoblast cells is unknown^20^.

In cells classically involved in cholesterol metabolism (i.e., hepatocytes), the abundance and function of lipoprotein receptors and cholesterol transporters are carefully regulated under exposure to increased levels of cholesterol, a phenomenon widely described^23^. Regarding the regulation of placental lipoprotein receptors and cholesterol transporters by increased maternal lipid levels, few studies have reported changes in the mRNA or protein level of SR-BI, LDLR, ABCA1 or ABCG1 in the whole placenta from women with increased levels of TC or TC and Tg^5,12^. No studies addressing functional changes of these proteins in MSPH have been reported.

Considering that the placenta is an organ with different cell types and that the receptors and transporters of cholesterol are differentially expressed, we aimed to determine the effect of MSPH on the protein abundance of LDLR, SR-BI, ABCA1 and ABCG1, on the processes of LDL and HDL uptake, on cholesterol efflux and on cholesterol content in the trophoblast cells.

## Material and Methods

### Study groups

Human placentas were obtained from 55 normal full-term pregnancies from the Hospital Clínico UC-CHRISTUS (HCUC, Chile, n = 43) and the Lindenhofgruppe (Switzerland, n = 12). The investigation conformed to the principles outlined in the Declaration of Helsinki. Ethics approval was obtained from the Faculty of Medicine of the Pontificia Universidad Católica de Chile (PUC, ID 11–066) and from the Canton of Bern, Switzerland (Basec Nr. 2016-00250). Informed consent and clinical data from patients were obtained as previously described^9^. General maternal (i.e., age, height, weight, blood pressure and glucose levels) and neonatal (i.e., sex, gestational age, weight and height) variables were obtained from the clinical records. In maternal and umbilical cord blood, the levels of TC; high-density lipoprotein (HDL), LDL and very-low-density lipoprotein (VLDL) cholesterol; and triglycerides (Tg) were determined at term of pregnancy (third trimester).

Pregnant women with TC < 280 mg/dL were included in the MPH group, and those with TC ≥ 280 md/dL in the MSPH group. The cut-off value for MSPH reflected values at which human fetoplacental endothelial and vascular dysfunction have been previously reported^1,2,4,5,7–9,24^. The exclusion criteria were maternal obesity at term of pregnancy, pre-gestational and gestational diabetes, preeclampsia, intrauterine growth restriction, foetal malformations and other maternal pathologies as described previously^9^.

Efflux assays to HDL and ApoA-I were performed on the samples from Chile and Switzerland. All other determinations were performed only in samples from Chilean women.

### Determination of maternal cholesterol and triglyceride levels

TC, HDL, LDL, VLDL and Tg levels were determined in maternal blood obtained from brachial venous blood and in umbilical cord blood at the time of delivery as described^9^. Lipid determination was performed in the Clinical Laboratory of the HCUC via standard enzymatic-colorimetric assays as previously described^9^. TC concentrations in samples from women in Switzerland were measured by using enzymatic kits purchased from Biomérieux.

### Primary human trophoblast (PHT) culture

PHT was obtained from the isolation of term cytotrophoblast from fresh placental villus tissue. After isolation, the cells were cultured until 72 h to obtain differentiated syncytiotrophoblast as described^25,26^. In brief, after rinsing in saline solution (10 mmol/L Hepes, pH 6.95 plus 0.9% p/v NaCl), the placental tissue was carefully minced, and 30 g was digested three times (30 min, 37 °C) in saline Hanks/HEPES solution (HBSS; mmol/L 5.37 KCl; 0.44 KH_2_PO_4_; 136.9 NaCl; 0.34 Na_2_HPO_4_; 5.55 glucose; 100 HEPES; 1.26 CaCl_2_; 0.81 MgSO_4_·7H_2_O; pH 7.4) plus DNase I (Worthington, USA) (total activity: 60.000, 40.000 and 30.000 kilounits, respectively, for each digestion) and trypsin (Thermo Fisher Scientific, USA) (total activity: 615.000, 410.000 and 315.000 BAEE units, respectively, for each digestion). After each digestion step, the solution was filtered. The digestion was stopped with newborn calf serum (final concentration: 20%) and centrifuged (975 RCF, 20 °C, 10 min). The cellular pellets were resuspended in DMEM and separated by centrifugation (1500 RCF, 20 °C, 20 min) in a Percoll gradient (10–70%) (GE Healthcare, USA). Cytotrophoblast cells were obtained from gradient fractions between 35 and 55%. Cells were plated at a density of 100,000 cells/cm^2^ in DMEM/F12 culture medium supplemented with foetal bovine serum (FBS, 10%) and 100 U/ml penicillin-streptomycin under standard conditions (37 °C, 5% CO_2_) for 72 h. All the supplies for culture medium were from Thermo Fisher Scientific (USA). The purity of the PHT culture (93–99%) was determined by staining the cells with the specific markers anti-cytokeratin 7 (CK7), anti-vimentin, anti-E-cadherin, and/or anti-von Willebrand factor (vWF) (Novus Biologicals, USA) followed by flow cytometry analysis in FACSDiva (BD Biosciences, USA) as described^27^. The syncytialization was confirmed by visualization under the microscope and by determination of human chorionic gonadotrophin in the culture media by ELISA (R&D Systems, USA). All the experiments were started performed after 72 h of culture.

### Western blot

Placental sections were homogenized to obtain protein extracts. Samples were lysed in solution 1 (mmol/L 10 EDTA, 50 Tris-HCl, pH 8.3), mixed with an equal volume of solution 2 (4% SDS, 20% glycerol, 125 mmol/L Tris/HCl, pH 6.8), heated (50 °C, 10 min), sonicated (6 cycles, 10 s, 100 Watts, 4 °C), and spin down (15000 RCF, 20 min) as described^9,28^. PHT was lysed in protein extraction buffer (100 mmol/L NaCl, 0.5% Triton X-100, 1% SDS, 50 mmol/L Tris/HCl, pH 7.4) containing a mixture of protease inhibitors. Extracts were sonicated, and the protein content was determined with the bicinchoninic acid (micro BCA) Protein Assay Kit (Thermo Fisher Scientific, USA) as described^28^.

Proteins were separated as described^9^ by polyacrylamide gel electrophoresis in denaturing and reducing conditions, transferred to polyvinylidene difluoride membranes and later probed with primary rabbit polyclonal anti-LDLR, anti-apolipoprotein B 100 (ApoB), anti- apolipoprotein A-I (ApoA-I) (Abcam, UK), anti-SR-BI, anti-ABCA1 and anti-ABCG1 (Novus Biological, USA), (1:1000, 18 h, 4 °C) and mouse monoclonal anti-3-hydroxy-3-methylglutaryl-CoA reductase (HMGCR, 1:500, 18 h, 4 °C) (Santa Cruz Biotechnology, USA) and anti-β-actin (1:5000, 1 h, room temperature) (Sigma-Aldrich, USA) antibodies. After wash the membranes were incubated (1 h, room temperature) with secondary horseradish peroxidase-conjugated goat anti-rabbit or anti-mouse antibody (Thermo Fisher Scientific, USA) as described^9^. Proteins were detected by enhanced chemiluminescence and quantified by densitometry. Uncropped blots are in Supplementary Data.

### Immunofluorescence

Formalin-fixed placental biopsies (10% buffered formalin solution, 24 h, 4 °C) were processed for routine paraffin embedding and sectioning (5 μm) for histological analysis as described^9,28^. For immunolocalization, the sections were dewaxed and rehydrated by serial incubations in ethanol (100, 98, 75, 50%, 5 min). Sections were then boiled in citrate buffer (10 mmol/L, 20 min, pH 6.0), rinsed in phosphate buffer solution (PBS, mmol/L: 130 NaCl, 2.7 KCl, 0.8 Na_2_HPO_4_, 1.4 KH_2_PO_4_, pH 7.4), and incubated (1 h, room temperature) in blocking buffer (100 mmol/L NaCl, 0.05% Triton X-100, 5% bovine serum albumin (BSA), 50 mmol/L Tris/HCl, pH 7.5). Placental sections were incubated (18 h, 4 °C) with the primary antibodies for ferroportin-1 (FPN-1, syncytiotrophoblast basal membrane marker), placental alkaline phosphatase (PLAP, syncytiotrophoblast apical membrane marker), CD31 (endothelial marker), LDLR, SR-BI, ApoB, ApoA-I, ABCA1, and ABCG1 and/or mouse monoclonal anti-CK7 (Sigma-Aldrich, USA) in blocking buffer (1:30). After rinsing twice with blocking buffer, the secondary antibodies Alexa Fluor 488-conjugated goat anti-rabbit (H + L, λexc/λem: 495/568 nm, 1:1000 dilution) and Alexa Fluor 568-conjugated goat anti-mouse IgG (H + L, λexc/λem: 578/603 nm, 1:1000 dilution) (Thermo Fisher scientific, USA) in blocking buffer containing 0.1 μg/mL DAPI (4',6-Diamidino-2-Phenylindole, Dihydrochloride) (Invitrogen, USA) were added as decribed^28^. Tissue sections were coverslipped and incubated for 1 h at room temperature. After rinsing twice with PBS (room temperature), the coverslips were examined in a Nikon Eclipse C2 confocal microscope. Images were processed with ImageJ version 1.48 (NIH, USA).

### Lipoprotein isolation and labelling

For uptake and efflux assays, lipoproteins from non-pregnant donors were isolated by ultracentrifugation as described^29^. Briefly, serum was obtained, and sucrose (final concentration: 10%), EDTA (10 mmol/L, pH 7.4), aprotinin (2 μg/mL) and phenylmethylsulfonyl fluoride (PMSF, 1 mmol/L) were added. The serum density was adjusted with KBr to 1.24 g/mL. Over the samples (1.7 mL), PBS (3.3 mL, density: 1.006 g/mL) was added to generate a density gradient by ultracentrifugation (rotor SW55Ti, 287000 RCF, 15 °C, 4 h). The bands corresponding to LDL, HDL and the lipoprotein-depleted serum (LPDS) were isolated from the gradient. After dialysis in saline solution (mmol/L 150 NaCl, 0.34 EDTA, pH 7.4, 4 °C, 48 h), LDL and HDL were stored at 4 °C in a sealed tube saturated with nitrogen. The protein concentration was determined as described in the western blot section. The correct isolation of lipoproteins was determined by SDS-PAGE separation followed by Coomassie R-250 staining and western blot for ApoB and ApoA-I.

For uptake assays, LDL and HDL were labelled with the lipophilic fluorescent dye 1,1′-dioctadecyl-3,3,3′,3′-tetramethylindocarbocyanine perchlorate (DiI, Invitrogen, USA) as described^30,31^. LDL or HDL (1 mg) was mixed with LPDS (2.5 mg/mL) and incubated with DiI (3 mg/mL in DMSO, 15 h, 37 °C). Subsequently, the reaction mixture was centrifuged (400 RCF, 20 °C, 5 min), and the density of the supernatant was adjusted to 1.24 g/mL with KBr. LDL-DiI and HDL-DiI were ultracentrifuged, isolated, dialyzed and stored as described in the previous paragraph.

### Uptake assays

Uptake of increased concentrations of LDL-DiI (0–200 μg/mL, 2 h, 37 °C) and HDL-DiI (0–50 μg/mL, 4 h, 37 °C) was measured in PHT cultured by 72 hours that were pre-incubated (overnight) in DMEM/F12 containing 5% FBS by modification of the protocols previously described^31–34^. After incubation, the cells were washed with PBS-BSA-free fatty acid (FFA) (2 mg/mL), and fluorescence (λexc/λem: 550/595) was quantified (Infinite M200Pro, Tecan, Austria). Subsequently, the cells were lysed with 0.5 N KOH, and the concentration of proteins in each sample was determined using a Bradford reagent kit (Bio-Rad, USA). In addition, a standard curve of LDL-DiI and HDL-DiI (0–200 μg/mL) was prepared, and the fluorescence was measured. The uptake was expressed as ng lipoprotein/mg of cellular protein (ng HDL-DiI or LDL-DiI/mg protein). The values were adjusted to the Michaelis-Menten hyperbola, and the maximal velocity (V_max_) and apparent Michaelis-Menten constant (K_m_) were calculated as previously described^7^.

### Efflux assays

The efflux of [^3^H]-cholesterol from PHT to ApoA-I or HDL was determined with minor modifications as described^35,36^. In brief, PHT cultured by 72 hours that were pre-incubated for 24 h with DMEM/F12 containing 10% FBS supplemented with [^3^H]-cholesterol (0.5 μCi/mL). After incubation, the culture medium was removed, and the cells were washed with PBS supplemented with BSA-FFA (2 mg/mL). Subsequently, the cells were incubated with non-pregnant isolated HDL (0–50 μg/mL, 6 h, 37 °C) or ApoA-I (10 μg/mL, 6 h, 37 °C) (Sigma-Aldrich, USA). Subsequently, the culture medium was recovered, and the cells were lysed with KOH. Radioactivity was determined both in the culture medium and in cell lysates, and the efflux was estimated as the fraction of radioactive signal in the medium compared to the total signal in the medium and cells. The values were adjusted to the Michaelis-Menten hyperbola, and V_max_ and K_m_ were calculated as previously described^7^.

To determine the relative contribution of SR-BI to the efflux of cholesterol, an assay was performed by inhibiting the function of the receptor with the reported inhibitor BLT-1 (0–50 μmol/L, 6.5 h, 37 °C) starting 30 min before HDL incubation^19,37^. As a control, parallel assays were performed to determine the reported effect of BLT-1 as an inhibitor of SR-BI-mediated uptake of HDL. HDL-DiI uptake (50 μg/mL, 6 h, 37 °C) was determined as described in the previous section in the presence or absence of BLT-1 (10 μmol/L, 1 h before the uptake assay, 37 °C)^37^.

### Cellular cholesterol content

Folch extraction was performed on 70 μg of protein from PHT lysate as described^38^. Lysates were incubated with methanol/chloroform (1:2 v/v, 30 min, 50 °C). Then, 1 volume of water was added (18 h, 4 °C), and the reaction was centrifuged (750 RCF, 20 min, 4 °C). The methanol/water phase was removed, and the chloroform phase containing cellular cholesterol was recovered and completely evaporated under nitrogen. Cholesterol extracted from PHT was determined with the Amplex Red Cholesterol assay (Invitrogen, USA) in the presence or absence of the enzyme cholesterol esterase for determination of total (TC) and free cholesterol (FC), respectively. Cholesterol esters were determined as the difference between TC and FC. FC was also determined by Filipin staining (Sigma-Aldrich, USA) as described^39^. PHT cells were fixed with 4% paraformaldehyde, their autofluorescence was quenched with glycine in PBS (1.5 mg/mL, 20 min, 20 °C), and then they were incubated with Filipin (25 μg/mL, 30 min, 20 °C). Images were obtained in an EVOS FL Imaging System (Life Technologies, USA), and the fluorescence was quantified with ImageJ version 1.48 (NIH, USA).

### Statistical analysis

**Figure 1:**
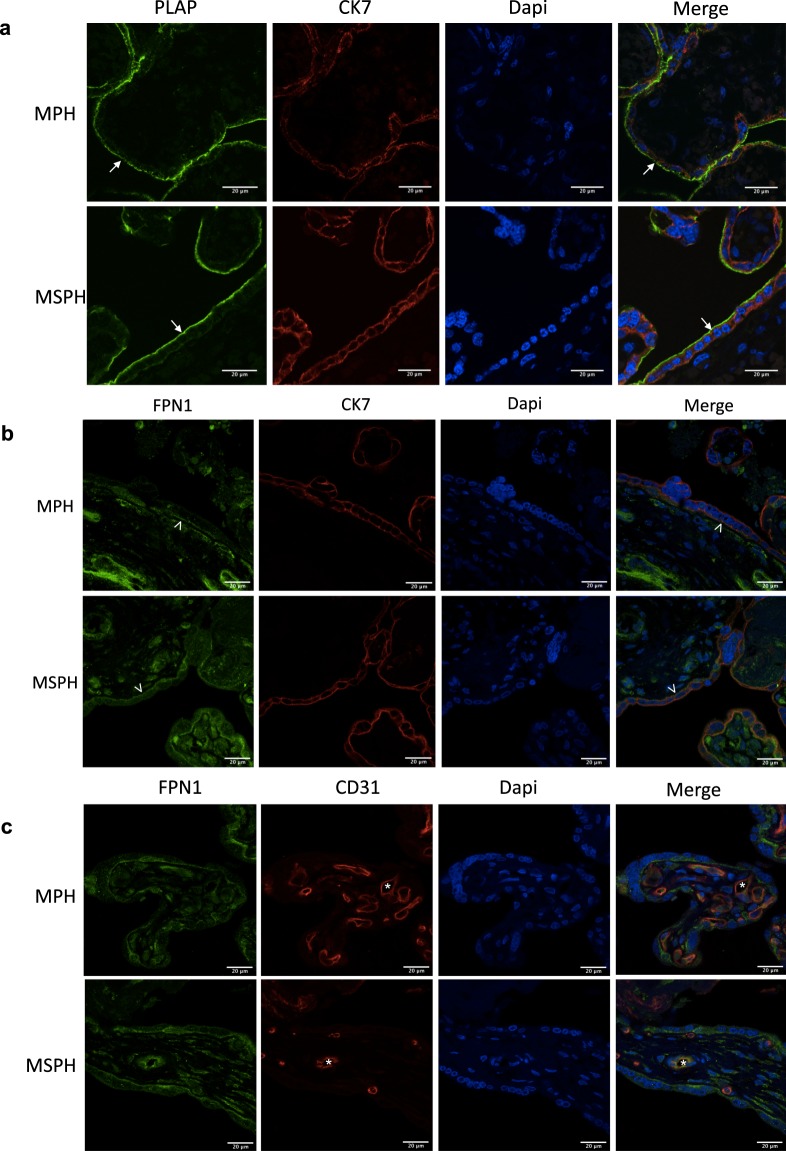
Syncytiotrophoblast and endothelial markers in the placenta. (**a**) Representative immunofluorescence for cytokeratin-7 (CK7, red), placental alkaline phosphatase (PLAP, green) and DAPI (blue) in placentas from MPH and MSPH pregnancies. (**b**) Representative immunofluorescence for CK7 (red), ferroportin-1 (FPN-1, green) and DAPI (blue) in placentas from MPH and MSPH pregnancies. (**c**) Representative immunofluorescence for CD31 (red), FPN-1 (green) and DAPI (blue) in placentas from MPH and MSPH pregnancies.

Values for maternal and neonatal characteristics are presented as the mean ± S.D as described^9^. For *in vitro* assays, the values are presented as the mean ± S.E.M., where *n* indicates the number of placentas or cell cultures used (MPH = 3–9 and MSPH = 3–9). Comparisons between two or more groups were performed by Student’s t-test or ANOVA (ANOVA used only in Fig. 1E). p < 0.05 was considered statistically significant. The software GraphPad Prism 7.0 (GraphPad Software Inc., USA) was used for data analysis.

## Results

### Maternal and neonatal variables in MPH and MSPH pregnancies

Maternal TC and LDL levels at term of pregnancy were higher in women from the MSPH group than the MPH group. No difference between groups was seen in the level of HDL, VLDL, triglycerides or the general maternal variables evaluated (Table 1).

**Table 1:** Clinical characteristics of pregnant women and newborns.

Variables	MPH (*n* = 27)	MSPH (*n* = 28)
**Maternal variables**		
Age (years)	32.6 ± 5.9 (23–45)	33.4 ± 5.4 (20–45)
Height (cm)	166.2 ± 6.2 (156–180)	161.1 ± 6.5 (148–173)
Weight (kg)		
Weight 1^st^ trimester (kg)	62.5 ± 5.5 (51–72)	59.1 ± 5.5 (48–69)
Weight 2^nd^ trimester (kg)	68.5 ± 5.5 (62–79)	64.7 ± 6.2 (55–74)
Weight 3^th^ trimester (kg)	73.8 ± 6.7 (62–92)	70.6 ± 6.5 (58–80)
Weight gain (kg)	11.3 ± 1 (11–20)	11.5 ± 1 (10–11)
BMI (kg/m^2^)		
BMI at 1^st^ trimester (kg/m^2^)	23.4 ± 1.7 (19.7–26.6)	22.4 ± 1.6 (19.7–25.7)
BMI at 2^nd^ trimester (kg/m^2^)	24.8 ± 1.8 (21.9–27.2)	22.1 ± 1.6 (22.6–28.1)
BMI at 3^th^ trimester (kg/m^2^)	27.2 ± 2 (19–30)	27.3 ± 1.9 (23–30)
Mean arterial pressure at delivery (mm Hg)	89 ± 8 (75–113)	90 ± 9.1 (74–102)
Basal glycaemia (mg/dL)	77.5 ± 5.3 (68–83)	79.6 ± 5.8 (68–85)
OGTT (mg/dL)		
Basal glycaemia	77.4 ± 3.7 (72–82)	78.8 ± 5.1 (73–89)
Glycaemia 2 hrs after glucose	90.6 ± 8.4 (80–102)	95.2 ± 12.9 (74–115)
Lipid levels at delivery (mg/dL)		
Total cholesterol	237.6 ± 26 (183–275)	328 ± 40* (289–441)
HDL	69.3 ± 18 (50–106)	79 ± 16 (51–110)
LDL	125.9 ± 26 (81–172)	192.4 ± 35* (125–283)
VLDL	46 ± 12 (30–77)	52 ± 12 (27–73)
Triglycerides	239 ± 63 (140–385)	270 ± 64 (133–390)
**Newborn variables**		
Sex (female/male)	13/14	15/13
Gestational age (weeks)	39.4 ± 1.1 (37–41)	39.1 ± 0.9 (37–42)
Birth weight (grams)	3427 ± 392 (2365–4340)	3459 ± 194 (2730–4440)
Height (cm)	50.1 ± 1.8 (47–55)	50.9 ± 1.9 (47–54)
Ponderal index (grams/cm^3^ × 100)	2.7 ± 0.2 (2–2.9)	2.6 ± 0.5 (2.2–2.8)
Lipid levels in umbilical blood (mg/dL)	n = 19	n = 24
Total cholesterol	58.5 ± 11.6 (46–77)	62.5 ± 13 (47–97)
HDL	28.8 ± 7 (20–44)	29.4 ± 6.3 (22–48)
LDL	22 ± 5 (16–35)	24.9 ± 6.3 (13–37)
Triglycerides	15.5 ± 4 (21–80)	14.4 ± 4 (20–66)

Women with maternal physiological (MPH, TC < 280 mg/dL) or supraphysiological hypercholesterolaemia (MSPH, TC ≥ 280 mg/dL) at delivery were included (see Methods). Weight and body mass index (BMI) were determined at trimester 1, 2 and 3. Blood pressure and lipid profiles were determined at delivery. OGTT, oral glucose tolerance test. HDL, high-density lipoprotein; LDL, low-density lipoprotein; and VLDL, very-low-density lipoprotein. For TC and LDL levels, *P < 0.0001 versus corresponding values in the MPH group. Data are presented as the mean ± S.D. (range).

Regarding the neonatal variables, no differences were found between the groups. Despite the increased levels of maternal TC and LDL, the neonatal lipid profile was similar between MPH and MSPH (Table 1).

### Syncytiotrophoblast and endothelial markers in the placenta

**Figure 2:**
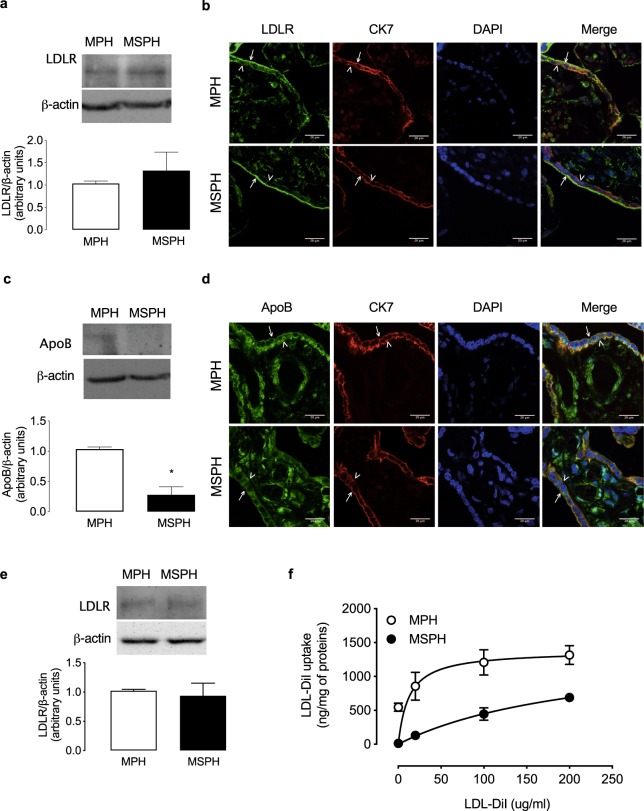
Placental LDL uptake. (**a**) Representative western blot for LDL receptor (LDLR) in placental homogenates from MPH (white) and MSPH pregnancies (black) (β-actin: internal control). (**b**) Representative immunofluorescence for LDLR (green), cytokeratin-7 (CK7, red) and DAPI (blue) in placentas from MPH and MSPH pregnancies. (**c**) Representative western blot for apolipoprotein B (ApoB) in placental homogenates from MPH (white) and MSPH pregnancies (black) (β-actin: internal control). *P < 0.0001. (**d**) Representative immunofluorescence for ApoB (green), CK7 (red) and DAPI (blue) in placentas from MPH and MSPH pregnancies. (**e**) Representative western blot for LDLR in primary human trophoblast (PHT) from MPH (white) and MSPH pregnancies (black) (β-actin: internal control). (**f**) Uptake o**f** labelled LDL-DiI (0–200 μg/ml, 2 hours, 37 °C) in PHT from MPH (white) and MSPH pregnancies (black). Arrows indicate the apical side of the syncytiotrophoblast, and arrowheads indicate the basal side. Scale bar corresponds to 20μm. *Significant difference versus MPH values. Values are mean ± S.E.M. (n = 3–6 per group for western blot, 3 for immunofluorescence and 9 for uptake assays).

The epithelial marker CK-7 was used to determine by immunofluorescence the syncytiotrophoblast layer in the whole placenta (Fig. 2A). In the same figure, PLAP was used to determine the apical membrane (arrows) of the syncytiotrophoblast layer. The basal membrane marker FPN-1 (arrowheads) was co-localized with and CK-7 (Fig. 2B) and also with the endothelial marker CD31 (asterisks, Fig. 2C).

### LDLR and LDL uptake

**Figure 3:**
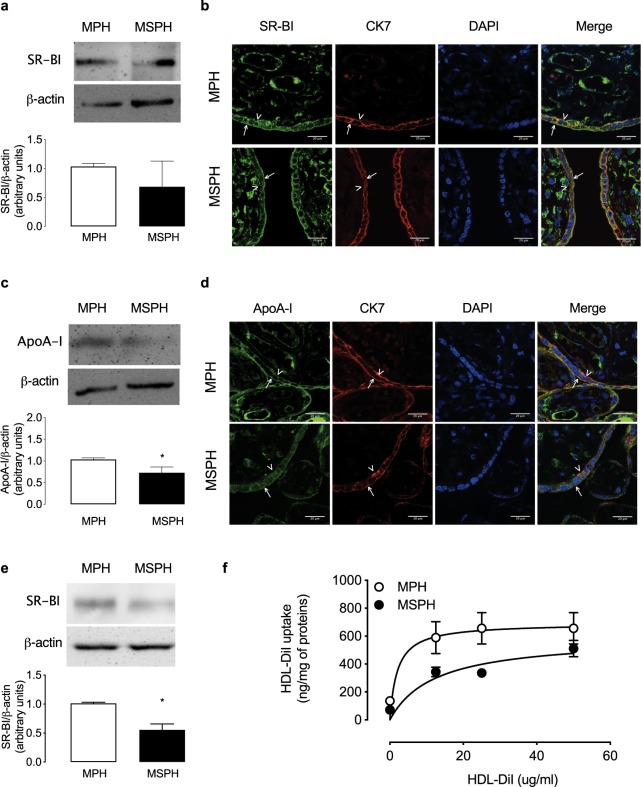
Placental HDL uptake. (**a**) Representative western blot for HDL receptor (SR-BI) in placental homogenates from MPH (white) and MSPH pregnancies (black) (β-actin: internal control). (**b**) Representative immunofluorescence for SR-BI (green), cytokeratin-7 (CK7, red) and DAPI (blue) in placentas from MPH and MSPH pregnancies. (**c**) Representative western blot for apolipoprotein A-I (ApoA-I) in placental homogenates from MPH (white) and MSPH pregnancies (black) (β-actin: internal control) *P = 0.049. (**d**) Representative immunofluorescence for ApoA-I (green), CK7 (red) and DAPI (blue) in placentas from MPH and MSPH pregnancies. (**e**) Representative western blot for SR-BI in primary human trophoblast (PHT) from MPH (white) and MSPH pregnancies (black) (β-actin: internal control) *P = 0.0014. (**f**) Uptake of labelled HDL-DiI (0–50 μg/ml, 4 hours, 37 °C) in PHT from MPH (white) and MSPH pregnancies (black). Arrows indicate the apical side of the syncytiotrophoblast, and arrowheads indicate the basal side. Scale bar corresponds to 20μm. *Significant difference versus MPH values. Values are mean ± S.E.M. (n = 6 per group for western blot, 3 for immunofluorescence and 9 for uptake assays).

The protein abundance and localization of the LDLR receptor was determined in whole placenta from MPH and MSPH pregnancies. In whole placental protein extracts, the LDLR levels were similar in MPH and MSPH (Fig. 3A). The analysis of the localization of the receptor showed that LDLR was localized in the syncytiotrophoblast layer of the placenta (determined by CK7 colocalization), suggesting localization mainly in the apical membrane (Fig. 3B, arrows). Interestingly, in MSPH placentas, LDLR was almost completely localized on the cell surface of syncytiotrophoblast, whereas MPH placentas the signal was determined at the cell surface and intracellularly (Fig. 2C, arrows). In addition to the LDLR levels, the protein abundance of the LDLR ligand ApoB in LDL particles was also determined. The protein abundance of ApoB determined by western blot was lower in MSPH placentas (Fig. 3C), and the staining for the protein in the syncytiotrophoblast also suggested lower levels compared to MPH placentas (Fig. 3D).

Subsequently, PHT were isolated from MPH and MSPH placentas to determine LDLR abundance and activity. As in the whole placenta, the protein abundance of LDLR was comparable in cells from both conditions (Fig. 3E). The uptake of LDL-DiI was lower in PHT from MSPH pregnancies (Fig. 3F). After adjustment to a Michaelis-Menten hyperbola, the kinetic parameters indicated that the estimated *V*_max_ was unaltered in MSPH cells, but *K*_m_ was higher compared to MPH cells (Table 2).

**Table 2:** Kinetic parameters for LDL- and HDL-DiI uptake and cholesterol efflux.

Parameter	MPH (*n* = 9)	MSPH (*n* = 9)	*P* value
*LDL-DiI uptake*			
*V*_max_ (ng/mg protein/hour)	1399 ± 336	1324 ± 51	0.5174
*K*_m_ (µg/mL)	12.7 ± 7.4	183.7 ± 9.8*	<0.0001
*HDL-DiI uptake*			
*V*_max_ (ng/mg protein/hour)	692 ± 144	523 ± 71.1*	0.0061
*K*_m_ (µg/mL)	2.04 ± 1.8	6.32 ± 3.9*	0.0087
*Cholesterol efflux*			
*V*_max_ (% efflux/hour)	37.8 ± 3.6	19.2 ± 8.2*	<0.0001
*K*_m_ (µg/mL)	13.4 ± 4.4	16.8 ± 8.2	0.2893

LDL-DiI and HDL-DiI uptake and cholesterol efflux to HDL were assayed (see Methods) in PHT isolated from placentas from pregnancies with maternal physiological (MPH, TC <280 mg/dL) or supraphysiological hypercholesterolemia (MSPH, TC ≥280 mg/dL) (see Methods). *V*_max_, maximal velocity; *K*_m_, apparent Michaelis-Menten constant. *Significant difference versus MPH values, *P* values are in the table. Values are mean ± S.D.

### SR-BI and HDL uptake

**Figure 4:**
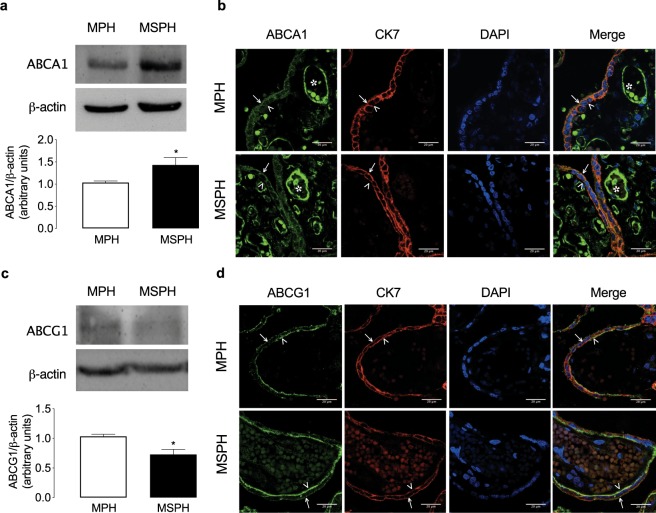
Placental protein abundance of cholesterol transporters. (**a**) Representative western blot for ATP-binding cassette transporter A1 (ABCA1) in placental homogenates from MPH (white) and MSPH pregnancies (black) (β-actin: internal control) *P = 0.042. (**b**) Representative immunofluorescence for ABCA1 (green), cytokeratin-7 (CK7, red) and DAPI (blue) in placentas from MPH and MSPH pregnancies. (**c**) Representative western blot for ATP-binding cassette transporter G1 (ABCG1) in placental homogenates from MPH (white) and MSPH pregnancies (black) (β-actin: internal control) *P = 0.006. (**d**) Representative immunofluorescence for ABCG1 (green), CK7 (red) and DAPI (blue) in placentas from MPH and MSPH pregnancies. Scale bar corresponds to 20μm. Arrows indicate apical side of the syncytiotrophoblast, arrowheads indicate basal side and asterisk endothelium. *Significant difference versus MPH values. Values are mean ± S.E.M. (n = 6 per group for western blot and 3 for immunofluorescence).

The protein abundance for the HDL receptor SR-BI was also determined in whole placenta from MPH and MSPH pregnancies. Protein levels (Fig. 4A) and placental *in situ* localization (Fig. 4B) of SR-BI were comparable in MPH and MSPH samples. SR-BI was determined in the syncytiotrophoblast layer (colocalization with CK-7), suggesting expression in apical (arrows) and basal membranes (arrowheads). In addition, the protein abundance of ApoA-I was determined. The levels of ApoA-I were lower in whole placenta protein extracts and in placental tissues from MSPH (Fig. 4C,D).

Subsequently, PHT were isolated from MPH and MSPH placentas to determine SR-BI abundance and activity. Unlike in whole-placental protein extracts, the protein abundance of SR-BI was lower in PHT from MSPH compared to MPH (Fig. 4E). The uptake of HDL-DiI was lower in PHT from MSPH pregnancies compared to MPH (Fig. 4F). The kinetic parameters indicated that *V*_max_ was lower and *K*_m_ was higher in MSPH cells compared to MPH (Table 2).

### Cholesterol transporters and cholesterol efflux

To determine the abundance of proteins related to cholesterol efflux, ABCA1 and ABCG1 protein levels were analysed. In whole-placental homogenates, the level of ABCA1 was increased and that of ABCG1 was reduced in MSPH compared to MPH (Fig. 1A,C). The immunofluorescence of the tissue suggested that ABCA1 was localized in the endothelium and both the apical (arrows) and basal membranes (arrowheads) of the syncytiotrophoblast layer (Fig. 4B). ABCG1 was mainly localized in the basal membrane (arrowheads, Fig. 1D). The signal for ABCA1 was higher in the endothelium of MSPH placentas (Fig. 1B).

**Figure 5:**
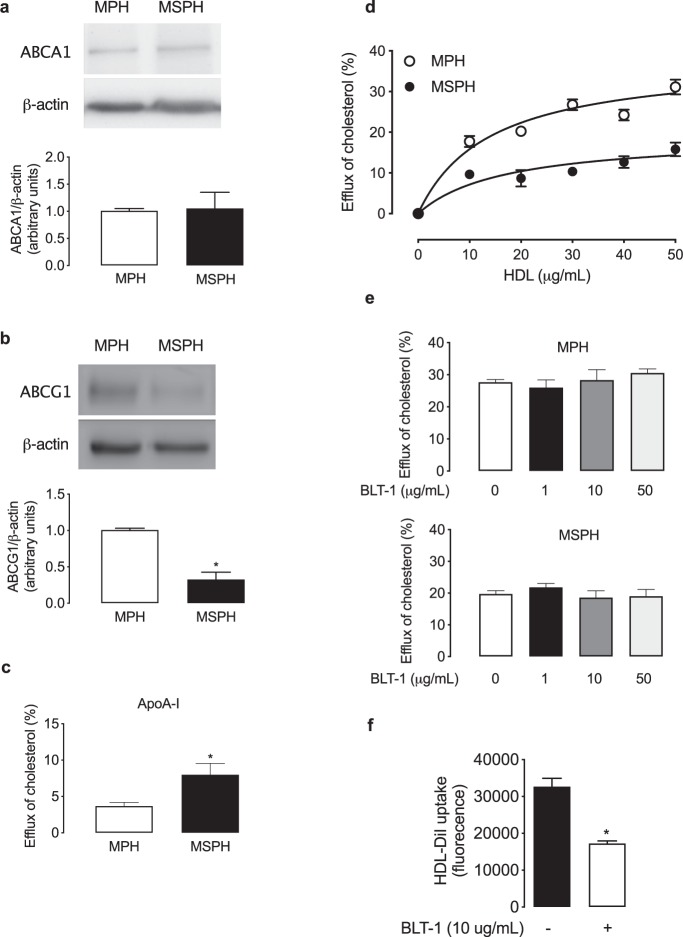
Placental cholesterol efflux. (**a**) Representative western blot for ATP-binding cassette transporter A1 (ABCA1) in primary human trophoblast (PHT) from MPH (white) and MSPH pregnancies (black) (β-actin: internal control). (**b**) Representative western blot for ATP-binding cassette transporter G1 (ABCG1) in PHT from MPH (white) and MSPH pregnancies (black) (β-actin: internal control) *P < 0.0001. (**c**) Efflux of cholesterol to ApoA-I (10 μg/ml, 6 hours, 37 °C) in PHT from MPH (white) and MSPH pregnancies (black) *P = 0.0026. (**d**) Efflux of cholesterol to HDL (0–50 μg/ml, 6 hours, 37 °C) in PHT from MPH (white) and MSPH pregnancies (black). (**e**) Efflux of cholesterol to HDL (50 μg/ml, 6 hours, 37 °C) in the absence or presence of the SR-BI inhibitor BLT-1 (0–50 μmol/L, 1 hour) in PHT from MPH and MSPH pregnancies. (**f**) Uptake of labelled HDL-DiI in the absence or presence of the SR-BI inhibitor BLT-1 (10 μmol/L, 1 hour) in PHT from MPH pregnancies *P = 0.0062. *Significant difference versus MPH values. Values are mean ± S.E.M. (n = 8 per group for western blot and 4–9 for efflux and uptake assays).

In isolated PHT, the protein abundance of ABCA1 was similar, while ABCG1 was reduced in cells from MSPH compared to MPH (Fig. 5A,B).

Cholesterol efflux to ApoA-I was increased in PHT from MSPH compared to MPH (Fig. 5C). The efflux of cholesterol to HDL was reduced in PHT isolated from MSPH compared to MPH placentas (Fig. 5D). The kinetic parameters for the efflux to HDL indicated that *V*_max_ was lower and *K*_m_ was similar in MSPH cells compared to MPH (Table 2).

The possible contribution of SR-BI to the HDL efflux was determined by pharmacological inhibition of SR-BI by BLT-1. The inhibition did not reduce cholesterol efflux to HDL (Fig. 5E). Considering the absence of changes with the inhibitor, we tested if BLT-1 was properly working as an inhibitor of SR-BI function. Therefore an uptake assay was done as a control of inhibition by the molecule. BLT-1 reduced the uptake of DiI-HDL by ∼50% (Fig. 5F).

### Cellular cholesterol levels

**Figure 6:**
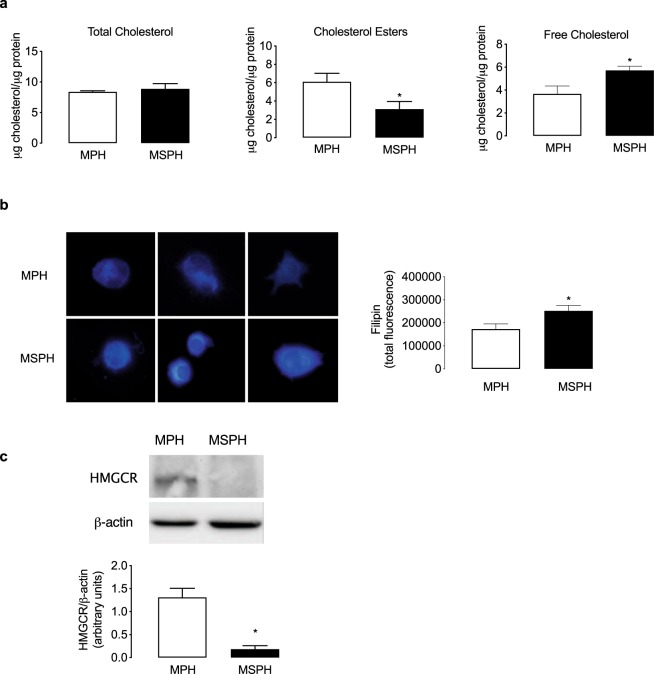
Cellular cholesterol levels in primary human trophoblast cells (PHT). (**a**) Total cholesterol, cholesterol esters (*P = 0.0042) and free cholesterol (*P = 0.0023) were determined enzymatically in PHT from MPH (white) and MSPH pregnancies (black). (**b**) Representative images (left) and quantification (right) for filipin staining of free cholesterol (25 μg/ml, 30 min, 20 °C) in PHT from MPH (white) and MSPH pregnancies (black) *P = 0.0023. (**c**) Representative western blot for HMGCR in PHT from MPH (white) and MSPH pregnancies (black) (β-actin: internal control) *P = 0.0061. *Significant difference versus MPH values. Values are mean ± S.E.M. (n = 5 per group for enzymatical assays and 3 for filipin staining and western blot).

Considering the changes in cholesterol uptake and efflux, the intracellular levels of total cholesterol, cholesterol esters and free cholesterol were determined in PHT from MPH and MSPH placentas. As shown in Fig. 6A, the levels of TC were comparable between both conditions. However, in PHT from MSPH, the level of cholesterol esters was reduced (Fig. 6A), and free cholesterol increased (Fig. 6A) compared to MPH, which was confirmed by filipin staining which only binds free cholesterol (Fig. 6B). To indirectly estimate the synthesis of endogenous cholesterol in PHT, the protein abundance of HMGCR was determined. In PHT from MSPH placentas, the level of the enzyme was reduced compared to cells from MPH (Fig. 6C).

## Discussion

In line with previous publications, this study confirmed that neonates from MSPH pregnancies (TC over a cut-off value of 280 mg/dL at term) have levels of TC, HDL and Tg similar to neonates from MPH women, suggesting a possible regulation of placental maternal-to-foetal cholesterol trafficking^7,12^. Since the placenta is the exchange barrier between maternal and foetal circulation, a possible regulation of placental maternal-to-foetal cholesterol trafficking across the placenta has been suggested^7,12^. This study shows for the first time that MSPH is associated with altered cholesterol trafficking in human placental syncytiotrophoblast, likely due to an imbalance between HDL and LDL uptake as well as cholesterol efflux.

Regarding the effects of maternal hypercholesterolaemia on human placental cholesterol trafficking, it was previously shown that in whole placenta from women with levels of TC over 280 mg/dL, the mRNA expression of LDLR and ABCG1 was higher and of SR-BI and ABCA1 unaltered compared to samples from women with TC levels lower this value^5^. In addition, placental protein extracts from pregnancies with levels of TC over 309 mg/dL and increased levels of triglycerides showed lower protein abundance for LDLR without changes in SR-BI compared to placentas with physiological levels of TC and triglycerides^12^. Despite these data, the effects of MSPH on the function of these proteins specifically in the placental layer responsible for the uptake of maternal lipoprotein-cholesterol and the initial cholesterol efflux to endothelial cells (i.e., syncytiotrophoblast) have not been addressed.

### LDLR and LDL uptake

LDLR was early described in the placenta^16,40^; however, only two reports showed the *in situ* localization of LDLR in placental tissue^17,41^. In agreement with both papers, our confocal microscopy images of the whole placenta showed that LDLR was expressed mainly in the placental syncytiotrophoblast layer and suggest that the localization could be predominantly in the apical membrane, which requires confirmation with specific markers for this syncytiotrophoblast membrane in the same section of tissue. In additional sections of the same placental tissue, was determined the localization of specifics markers of apical (PLAP) and basal membrane (FPN-1), supporting the findings described in the Fig. 3. Interestingly, our data showed that the protein abundance of LDLR was similar in MPH and MSPH placental homogenates. However, the distribution of the receptor was mainly on the surface of the syncytiotrophoblast layer in MSPH placentas, with a lower intracellular signal compared to that in MPH, suggesting that the endocytic activity of the receptor could be reduced. However, this hypothesis requires evaluation with detailed mechanistic studies, considering the diversity of possible involved proteins related to cellular LDLR trafficking^42,43^. Most of these proteins have been reported in the placenta but not yet functionally studied^44,45^. To indirectly evaluate the activity of LDLR, the levels of its ligand ApoB were analysed in MSPH placentas. The protein abundance, as determined by western blot, was lower in MSPH placentas, which was confirmed in the immunofluorescence assays. These findings suggest that in MSPH placentas, LDLR receptor levels are unaltered, but the localization and function of the receptor suggests reduced LDL uptake. Our results are different from those shown by Ethier-Chiasson *et al.*, who reported reduced protein abundance of LDLR in women with increased TC and Tg levels^12^. In contrast, our MSPH samples were from women with increased TC levels but not increased Tg levels compared to MPH samples, suggesting that the receptor could be differentially modulated by the maternal lipid profile.

To directly determine the function of LDLR, PHT cells were isolated from MPH and MSPH placentas. In concordance with the placental data, the protein abundance of the receptor was comparable in both conditions. In addition, the uptake of LDL-DiI was reduced in cells from MSPH. The analysis of the kinetic parameters showed similar *V*_*max*_ and increased *K*_*m*_, suggesting changes in the functional activity of the receptor more than changes in the number of receptors, which agrees with our results of protein expression.

### SR-BI and HDL uptake

The expression of SR-BI in isolated syncytiotrophoblast and placental endothelial cells has been previously reported^18–20,40^. However, only one report recently showed the localization of SR-BI in the syncytiotrophoblast layer of the placenta^46^. They suggest that SR-BI is mainly localized on the apical side of the trophoblast layer, although a proper marker of the trophoblast layer was not used in this paper. In our experiments on co-localization with CK7, we found that SR-BI was localized in the syncytiotrophoblast layer and possibly in both the apical and basal membranes. In additional sections of the same placental tissue (Fig. 2), was determined the localization of specifics markers of apical (PLAP) and basal membrane (FPN-1), supporting the findings described in the Fig. 4. This information suggests that the HDL receptor could participate in HDL cholesterol uptake but also that it is a possible mediator of cholesterol efflux (see next section).

In addition, and in accordance with a previous report^12^, SR-BI levels were similar in whole placentas from MPH and MSPH pregnancies. The placental localization of SR-BI was also comparable. Although classically SR-BI is recognized as a HDL receptor that mediates the selective uptake of the cholesterol esters bound to HDL and not the complete uptake of the HDL macromolecule^47^, Wadsack *et al*. showed that in the syncytiotrophoblast from term placentas, the uptake of HDL cholesterol is mediated in equal proportions by selective uptake and the complete uptake of the HDL^18^. Based on this evidence, we evaluated the levels of the HDL protein ApoA-I in the placental tissue as an indirect way to determine SR-BI activity. Interestingly, in MSPH placentas, the level of ApoA-I was reduced compared to MPH, indirectly suggesting that the placental uptake of HDL could be reduced. In addition, recently was reported that placenta secretes ApoA-I^48^, a process that also could be altered in MSPH placentas; however, require to be further studied.

To directly determine HDL uptake, PHT were isolated from MPH and MSPH placentas. The protein abundance of SR-BI as well as the HDL-DiI uptake was lower in MSPH cells. In accordance with the reduced protein abundance, the kinetic parameters indicated that the *V*_*max*_ for HDL-DiI uptake was reduced in cells from MPH. Additionally, the *K*_*m*_ was higher, suggesting that the number of receptors and their activity could be reduced.

In summary, we showed that the uptake of LDL-DiI and HDL-DiI are reduced in trophoblast cells from MSPH placentas. In the third trimester, foetal cholesterol is mainly synthesized by the foetal liver, and the uptake of cholesterol from the maternal lipoproteins is lower than in earlier periods of gestation. It has been suggested that at term, the placenta may play a protective role to prevent excessive cholesterol transport from the maternal to the foetal circulation^5,17,18^. Considering that MSPH women have increased circulating lipid levels and that the lipid levels in their neonates are comparable to those of MPH pregnancies, our findings of reduced maternal lipoprotein uptake by the syncytiotrophoblast are in accordance with that suggestion.

### Cholesterol transporters and cholesterol efflux

The efflux of cholesterol from trophoblast cells to acceptors such as HDL or ApoA-I has been previously reported. Classically, it has been described that the efflux to HDL is mediated by ABCG1 and to ApoA-I by ABCA1^19,20,48–50^. Our results of *in situ* localization are in agreement with previous papers describing ABCA1 in the syncytiotrophoblast layer of the placenta^20,49,51–53^. Although a proper marker for syncytiotrophoblast apical and basal membrane is required in the same tissue section, our findings are also in accordance with these reports and suggests a distribution of the transporter on the apical and basal sides of the trophoblast layer, indicating a possible role in the export of cholesterol to the maternal and also the foetal circulation. In additional sections of the same placental tissue (Fig. 2), was determined the localization of specifics markers of apical (PLAP) and basal membrane (FPN-1), supporting the findings described in the Fig. 4. Interestingly, western blot indicated a major signal for ABCA1 in MSPH placentas. Interestingly, western blot assay indicates a mayor signal for ABCA1 in MSPH placentas, a finding that by observation of the immunolocalization we associate with increased signal in the endothelium rather than in the trophoblasts. In the Fig. 2 is showed a proper marker of endothelium (CD31) in placental slides form the same placentas. However, specific endothelial markers in the same slide are required to confirm the spatial distribution of the endothelium. Increased ABCA1 levels in placental endothelial cells were recently described in gestational diabetes mellitus^54^, and other pregnancy metabolic disorders have been associated with maternal dyslipidaemia^55^. Our findings in PHT confirmed these observations in the whole placenta and showed that the levels of ABCA1 were similar in PHT from MSPH and MPH placentas.

Regarding ABCG1, our data suggest that the cholesterol transporter was mainly localized on the basolateral side of the trophoblast layer, as previously described^20^, suggesting a possible role in the export of cholesterol to the foetal circulation. In MSPH placentas, reduced levels of this transporter were determined by western blot, which was also confirmed in PHT cells from MSPH placentas.

In addition to the protein levels, the efflux of cholesterol to HDL and ApoA-I was determined in PHT. The efflux to exogenous ApoA-I was increased in PHT from MSPH compared to MPH. Considering the similar protein levels of ABCA1, these results suggest that the activity of the transporter could be increased in this condition of increased cholesterol availability, as described in other models, including endothelial cells from the placenta^19,23^. On the other hand, the efflux to HDL was decreased in PHT from MSPH pregnancies, which was associated with reduced protein levels of ABCG1. The kinetic parameters for efflux to HDL showed reduced V_*max*_ and comparable K_*m*_, suggesting that the changes could be related to lower levels of this transporter rather than changes in its activity.

The syncytiotrophoblast is a multinucleated and polarized tissue that presents an apical (in contact with the maternal circulation) and a basal membrane (in contact with the stroma and foetal endothelium). Therefore, the cholesterol efflux may occur to the maternal and/or the foetal side of this tissue, directionality that was not possible to be determined with our 2D model. Then, it is required to determine the efflux rates in cultured polarized PHT to improve our understanding of the directionality of the cholesterol efflux (to apical/maternal or basal/foetal)^48,50^. However, and based on: (1) the placental localization of ABCA1 (transport to ApoA-I) and ABCG1 (transport to HDL), (2) recent evidence showing that the efflux to ApoA-I in PHT is mainly to the apical side^48^, (3) that the expression of ApoA-I in the neonatal lipoproteins is very low compared to maternal concentration of this apolipoproteina^29^; we speculate that in PHT from MSPH placentas, the increased efflux to ApoA-I could be to the maternal circulation and the reduced efflux to HDL could be to the fetal circulation maybe as a possible regulation to control increased cholesterol flux to the foetal circulation.

In addition to the changes in the efflux rate in MSPH cells, the proportion of efflux to ApoA-I and HDL was different in PHT cells. In our model, the efflux to ApoA-I (10 µg/mL, 6 h) was ∼4%, compared with ∼30% to HDL (50 µg/mL, 6 h). This finding was different from those described by Aye *et al*., who showed comparable percentages of efflux (∼10%) to ApoA-I (20 µg/mL, 6 h) and HDL (50 µg/mL, 6 h) in PHT from normal pregnancies^20^. In addition, our results were comparable to the results recently reported by Harmon *et al*. in PHT from normal pregnancies^49^ showing efflux to ApoA-I (30 µg/mL, 6 h) of ∼5%, compared with ∼25% to HDL (50 µg/mL, 6 h)^49^. Harmon *et al*. did not discuss the differences in the rate of efflux to HDL and ApoA-I, even considering the reduced expression of ABCA1 reported in their study or the expression and function of ABCG1 previously described in PHT. In line with those and our results, we propose that in PHT, the contribution of ABCG1 to the total efflux could be higher than that of ABCA1, which requires confirmation of the inhibition of ABCA1- and ABCG1-mediated efflux in our system.

Besides ABCA1 and ABCG1, another transporter that could contribute to cholesterol efflux in PHT is SR-BI, which has an active role in cholesterol efflux in cells such as macrophages^21,22^. In endothelial cells from the placenta, SR-BI does not contribute to cholesterol efflux^19^. Despite that and considering that the receptor is described in the placental trophoblast cells^18,20^, its participation in cholesterol efflux has been only suggested, and its real contribution to the process is unknown^20^. To determine the possible contribution of SR-BI to the efflux of cholesterol, the receptor was inhibited with BLT-1, and the efflux was determined as described in placental endothelial cells^19,20^. The inhibition of SR-BI with BLT-1 was not associated with changes in cholesterol efflux, suggesting for the first time that, as in placental endothelial cells, SR-BI does not participate in cholesterol efflux in PHT cells. Nevertheless, we suggest that it is necessary to determine the SR-BI-mediated efflux in polarized PHT to exclude the possibility of compensatory changes in the apical and basal efflux in the presence of BLT-1. As a control of BLT-1 activity in our PHT model, the SR-BI-mediated uptake of HDL was assayed in the presence of the inhibitor. Our results showed that BLT-1 reduced HDL-DiI uptake by ∼50%, a value of inhibition lower than reported in non-placental cells with the same inhibitor^31^ but higher than reported with SR-BI blockers antibodies in the same cells^18^, confirming that BLT-1 is properly working.

In summary, we suggest that in PHT, cholesterol is delivered by efflux to acceptors such as HDL and ApoA-I; however, HDL is the acceptor with a predominant contribution to cholesterol export in these cells, a process that could be mediated by ABCG1 and ABCA1 and not by SR-BI. Regarding MSPH, PHT isolated from placentas with this maternal condition showed reduced efflux to HDL and increased efflux to ApoA-I, suggesting alterations in the release of cholesterol to the maternal or foetal circulation.

### Cellular cholesterol levels

Considering the reduced uptake of LDL and HDL as well as the changes in cholesterol efflux in PHT cells from MSPH pregnancies, the levels of intracellular cholesterol were determined in these cells. Although the levels of total cholesterol were similar in PHT from MPH and MSPH, the levels of free cholesterol were increased in MSPH compared to MPH. To indirectly determine if MSPH induces increased cholesterol synthesis, the level of HMGCR, the rate-limiting enzyme for cholesterol synthesis, was estimated. In PHT from MSPH placentas, the protein abundance of the enzyme was reduced, suggesting that in our model no increased cholesterol synthesis occurred. We propose that the mechanism associated with free cholesterol esterification (i.e., acyl-coenzyme A:cholesterol acyltransferases, ACATs) could be altered in MSPH cells, which should be further investigated.

Classically, increased levels of free cholesterol lead to activation of the nuclear factors LXRs, which is associated with increased cholesterol efflux and inhibition of cholesterol synthesis and uptake^56^. Although in this work we did not directly evaluate the activation of LXR in MSPH placentas, we suggest that LXR would not be activated since we did neither find increased ABCA1/ABCG1 nor reduced LDLR protein abundance in those placentas or in isolated PHT. Although the activation of LXR in trophoblast cells has not been studied in detail, LXR activation has been associated with reduced cell differentiation and cell survival, depending on the source of the ligand (exogenous vs endogenous), both phenomena that were apparently unaltered in PHT from MSPH^20,49,57–60^. Consequently, a potential follow-up of this study could be to determine the activation of LXR as well as the levels of possible ligands such as oxysterols in PHT from MSPH. In the context of placental cells, in endothelial cells isolated from GDM placentas, it was confirmed that the activation of LXR by oxysterols was related to increased ABCA1 and ABCG1 expression and cholesterol efflux, which, unlike in PHT cells, was suggested as a mechanism to maintain a proper cellular function^54^, as described in other cellular models^61^.

Finally, although MSPH treatment or prevention studies are necessary to establish a causal effect, we describe here for the first time that MSPH is a maternal condition that impairs cholesterol trafficking in the syncytiotrophoblast, increasing the cellular levels of free cholesterol, which could be associated with changes in the placenta-mediated maternal-to-foetal cholesterol trafficking. The relevance of this regulation could be related to the fact that the cholesterol levels in the neonates from MPH and MSPH pregnancies are similar even when the maternal levels of lipids are increased in MSPH women.

## Supplementary information

Supplementary figures.

## Data Availability

The datasets generated during and/or analysed during the current study are available from the corresponding author on reasonable request.
