# Supplementary material for: Cholesterol uptake and efflux are impaired in human trophoblast cells from pregnancies with maternal supraphysiological hypercholesterolemia
Source: Sci Rep. 2020 Mar 24;10:5264. doi: 10.1038/s41598-020-61629-4 (PMC7093446; doi:10.1038/s41598-020-61629-4)
Supplement: Supplementary file 1 — Supplementary figures. [file 41598_2020_61629_MOESM1_ESM.pdf]

Cholesterol uptake and efflux are impaired in human trophoblast cells from pregnancies with maternal supraphysiological hypercholesterolemia

Bárbara Fuenzalida[1], Claudette Cantin[1], Sampada Kallol[2,3], Lorena Carvajal[1], Valentina Pastén[1], Susana Contreras- Duarte[1], Christiane Albrecht[2,3], Jaime Gutierrez[4], Andrea Leiva[1,4*]

[1]Division of Obstetrics and Gynaecology, School of Medicine, Faculty of Medicine, Pontificia Universidad Católica de Chile, Santiago, Chile.

[2]Institute of Biochemistry and Molecular Medicine, Faculty of Medicine, University of Bern, Bern, Switzerland.

[3]Swiss National Centre of Competence in Research, NCCR TransCure, University of Bern, Bern, Switzerland.

[4]School of Medical Technology, Health Sciences Faculty, Universidad San Sebastian, Santiago, Chile.

***Correspondence**: Dr. Andrea Leiva
Division of Obstetrics and Gynaecology
School of Medicine, Faculty of Medicine
Pontificia Universidad Católica de Chile
P.O. Box 114-D, Santiago 8330024, Chile.
Telephone: +562-23548116
E-mail: aaleiva@uc.cl

Supplementary figure 1

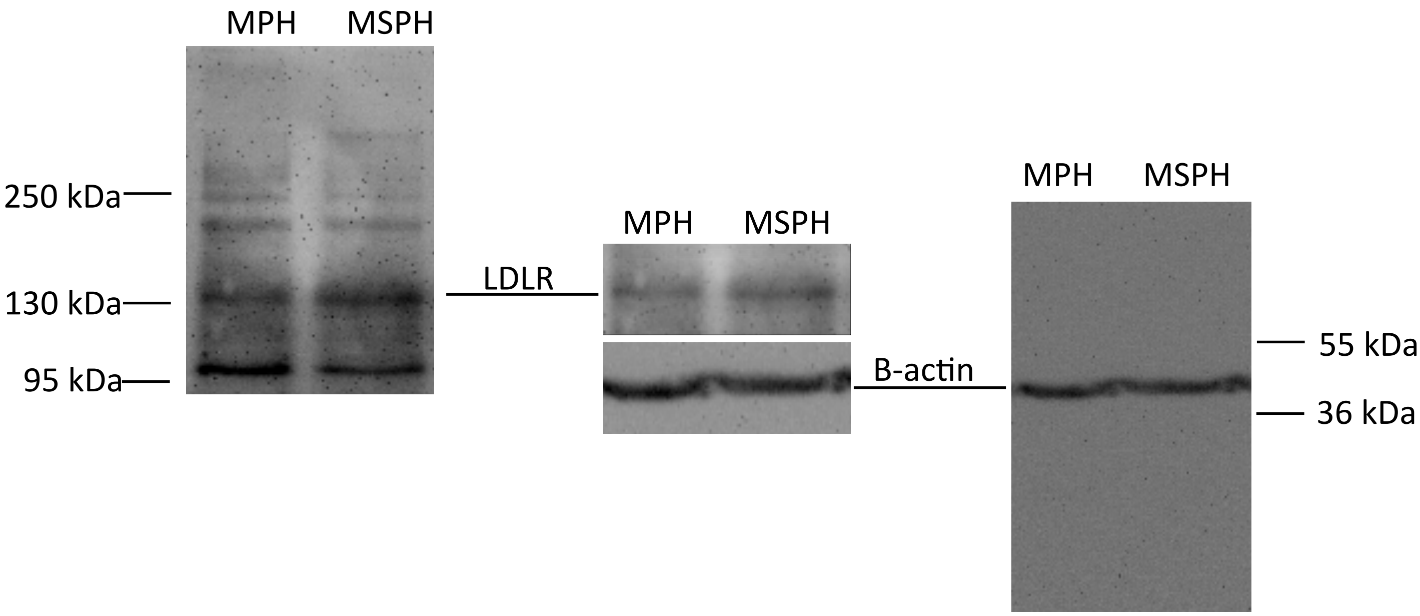

Supplementary figure 1: Corresponde to the uncropped image for figure 1A: LDLR in placental tissue (antibody: Ab52818, Abcam)

Supplementary figure 2

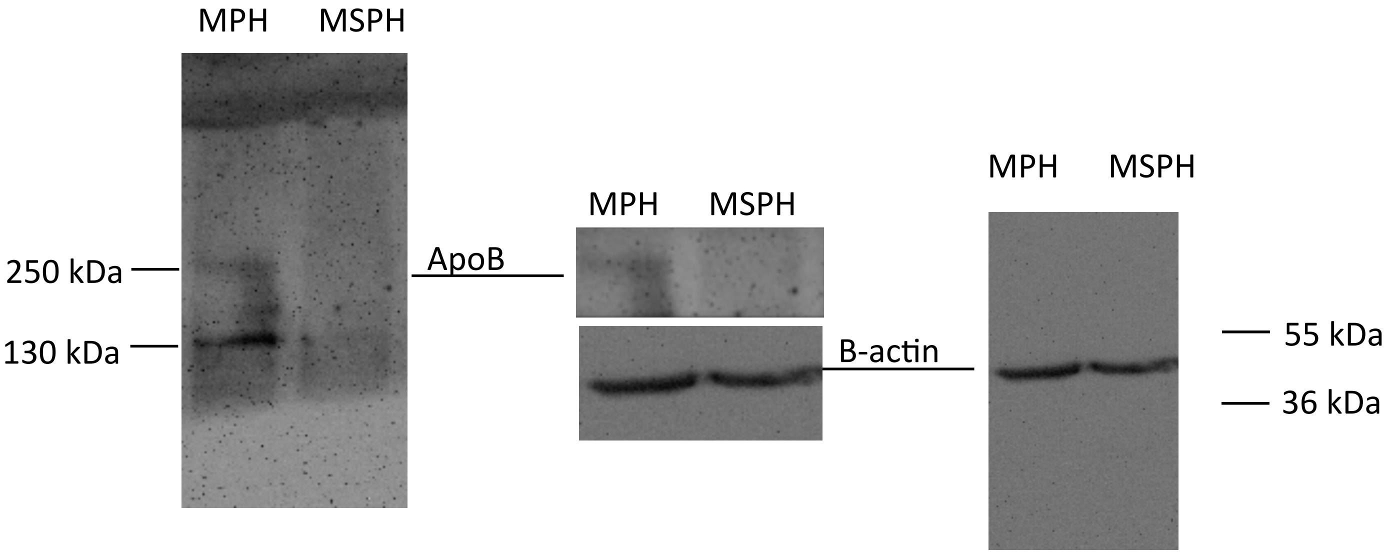

Supplementary figure 2: Corresponde to the uncropped image for figure 1C: Apolipoprotein B in placental tissue (antibody: Ab31992, Abcam)

Supplementary figure 3

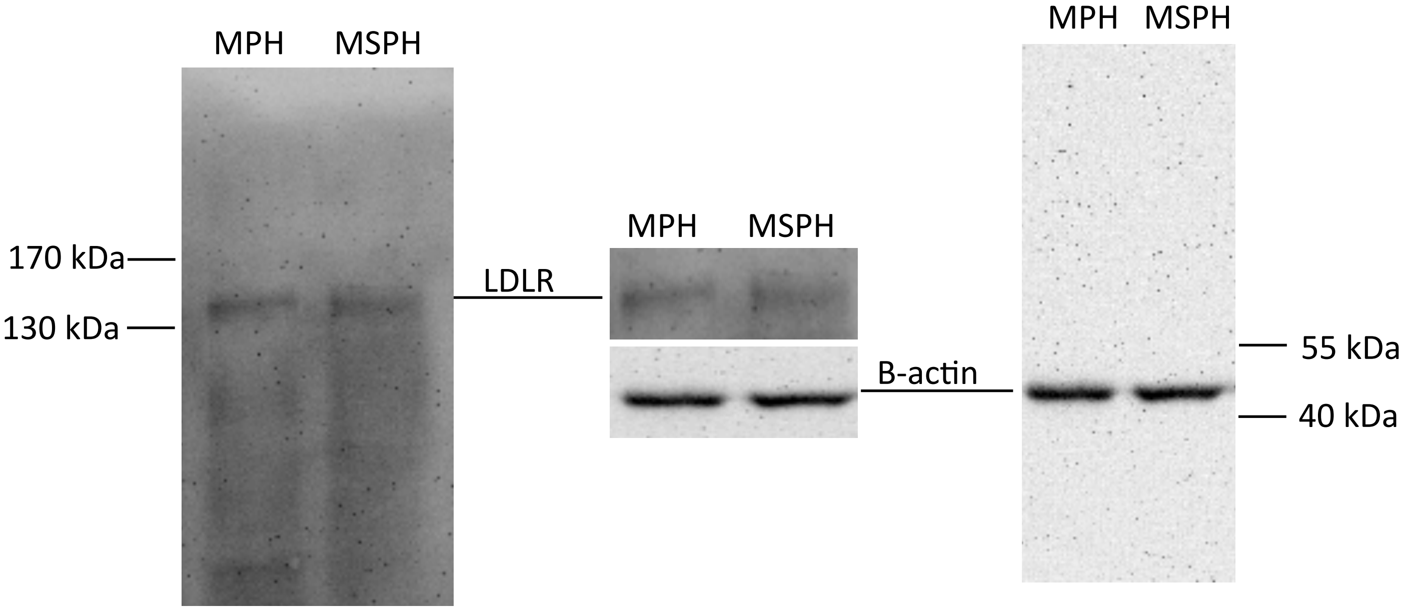

Supplementary figure 3: Corresponde to the uncropped image for figure 1E: LDLR in PHT cells (antibody: Ab52818, Abcam)

Supplementary figure 4

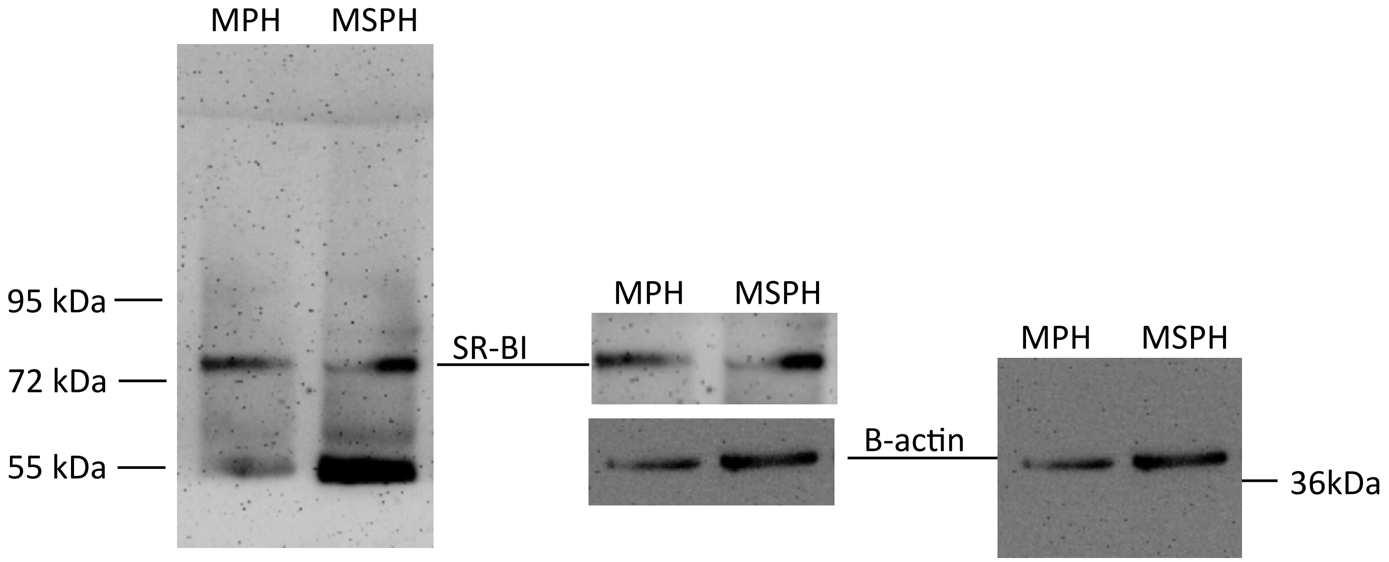

Supplementary figure 4: Corresponde to the uncropped image for figure 2A: SR-BI in placental tissue (antibody: NB400-101, Novus Biological)

Supplementary figure 5

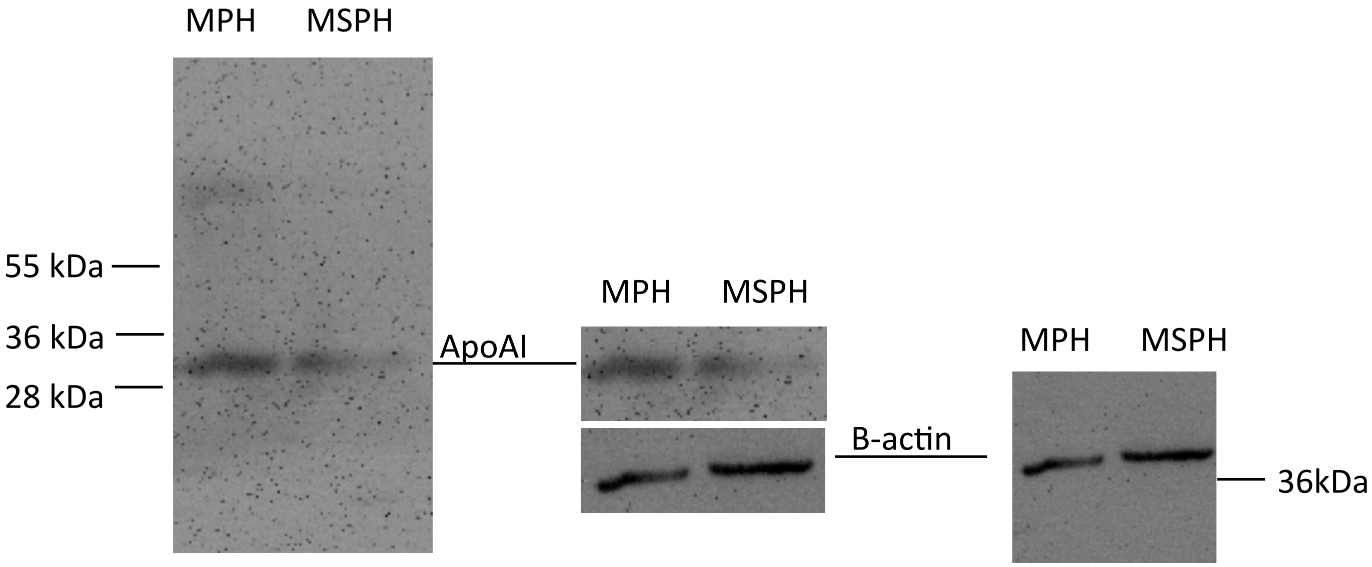

Supplementary figure 5: Corresponde to the uncropped image for figure 2C: Apolipoprptein A-I in placental tissue (antibody: Ab33470, Abcam)

Supplementary figure 6

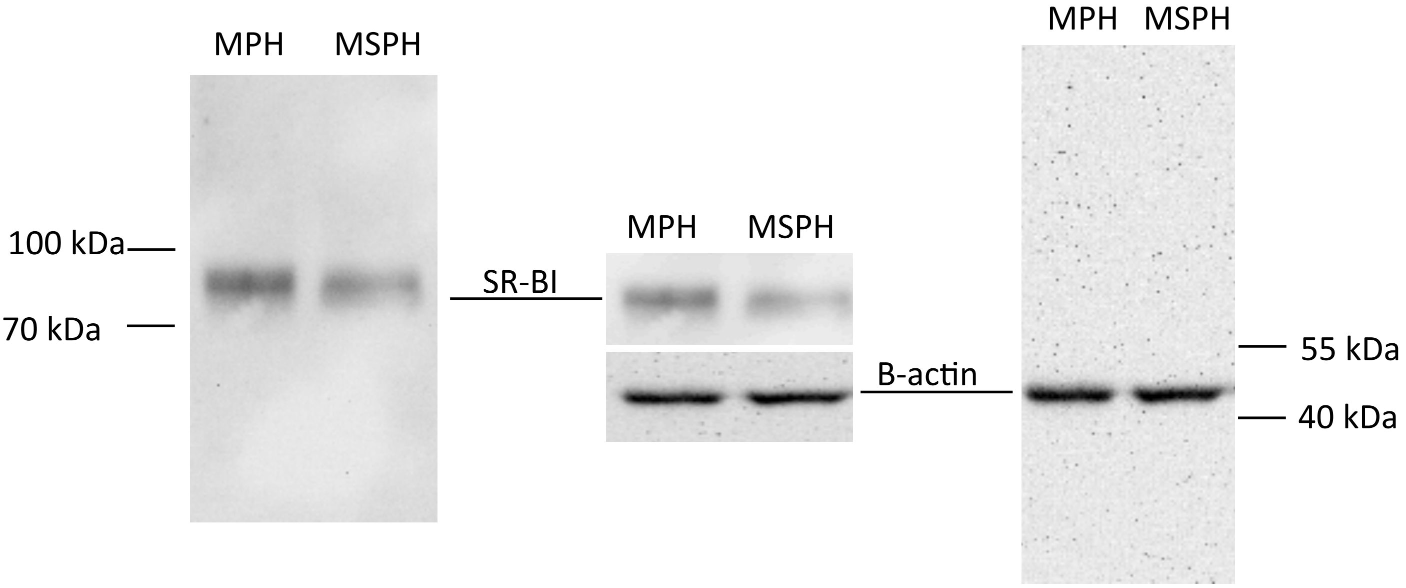

Supplementary figure 6: Corresponde to the uncropped image for figure 2E: SR-BI in PHT cells (antibody: NB400-101, Novus Biological)

Supplementary figure 7

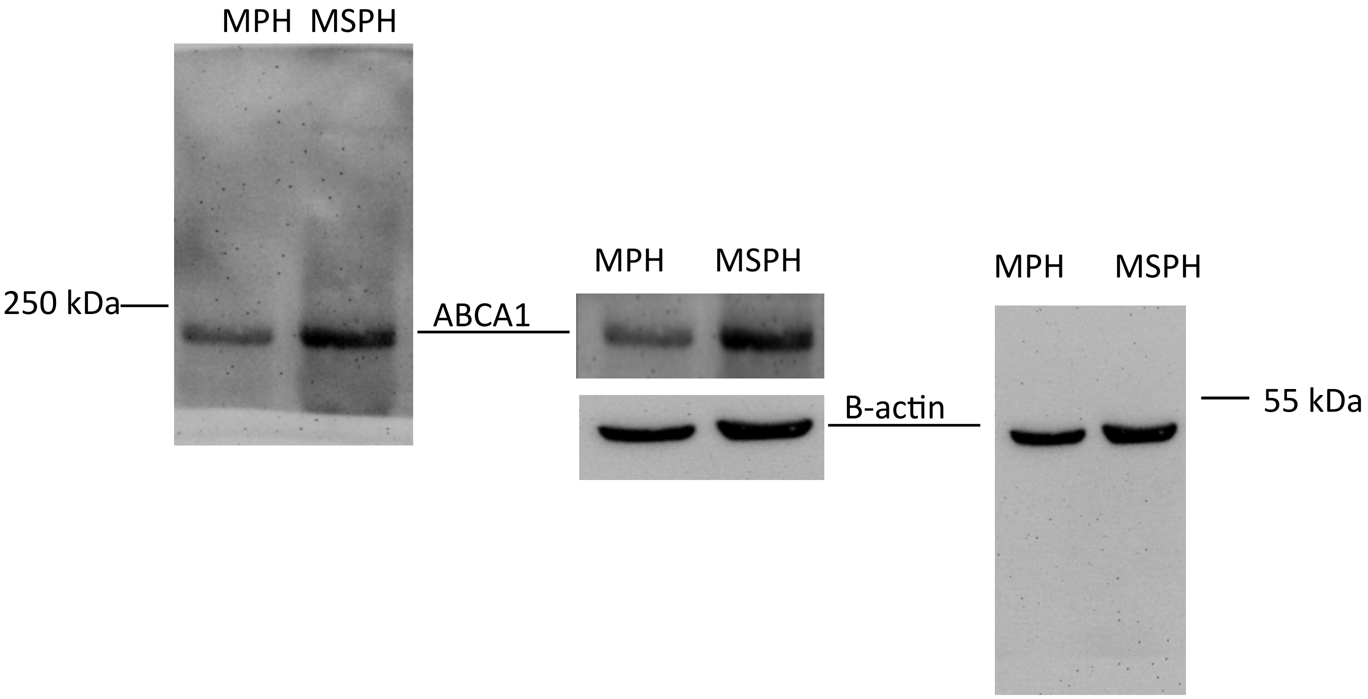

Supplementary figure 7: Corresponde to the uncropped image for figure 3A: ABCA1 in placental tissue (antibody: NB400-105, Novus Biological)

Supplementary figure 8

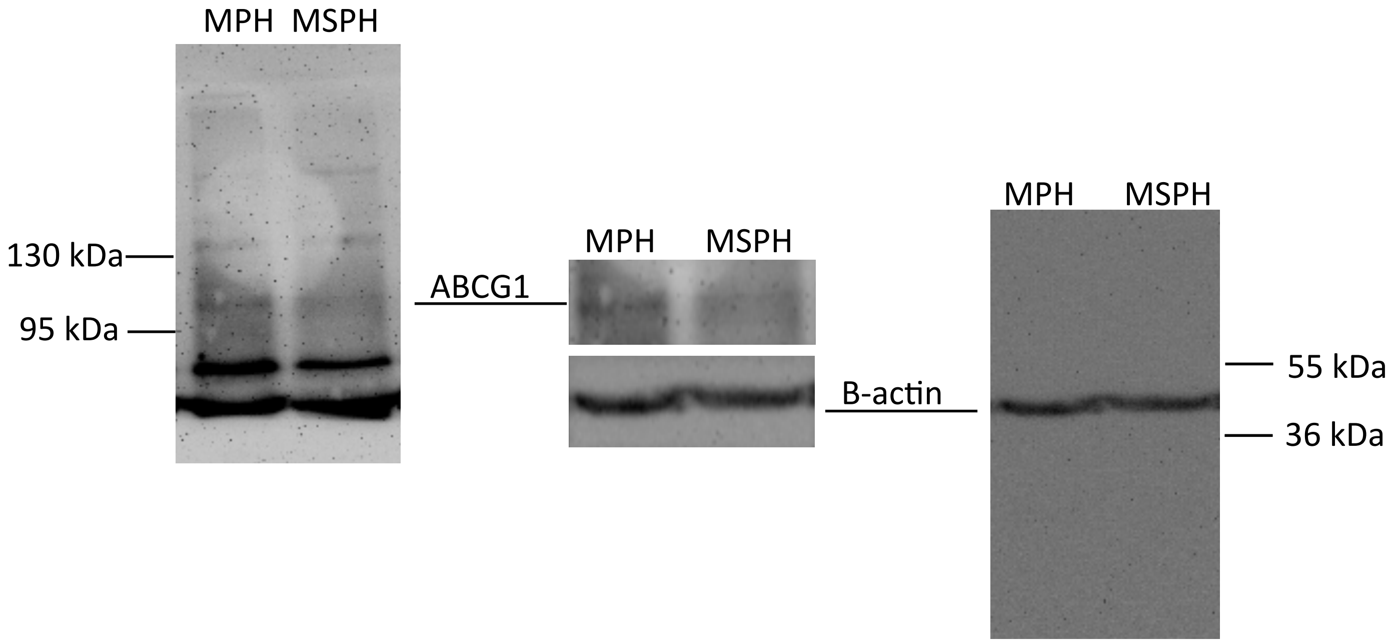

Supplementary figure 8: Corresponde to the uncropped image for figure 3C: ABCG1 in placental tissue (antibody: NB400-132, Novus Biological)

Supplementary figure 9

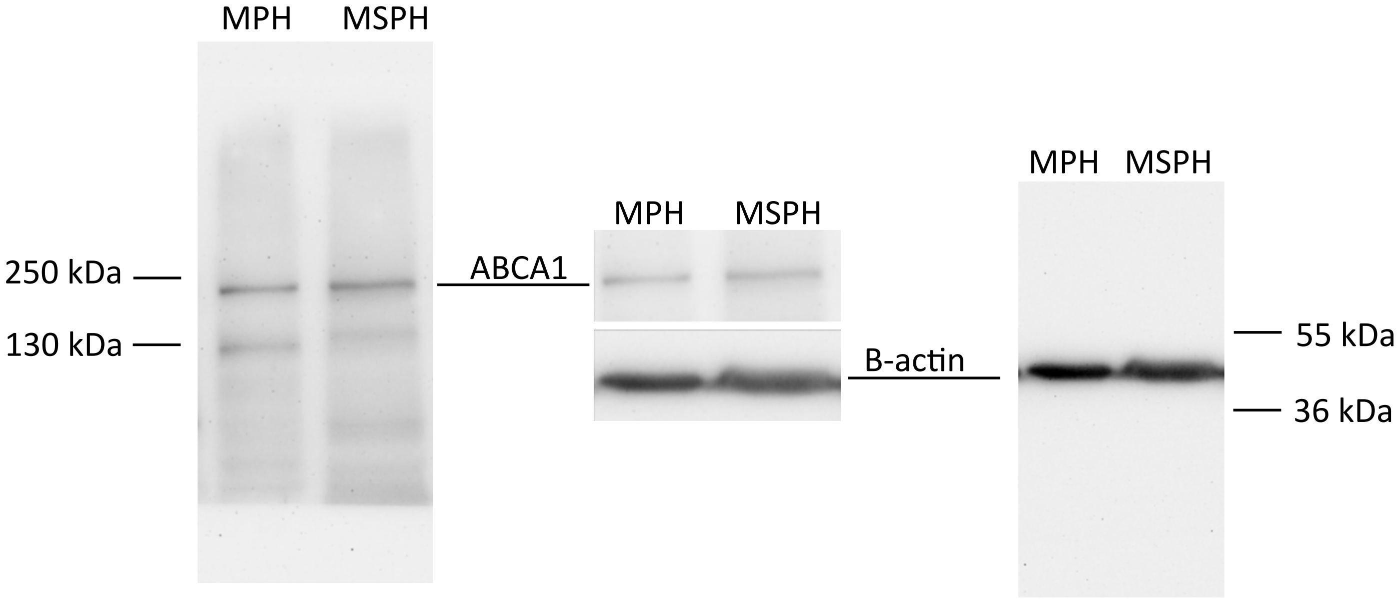

Supplementary figure 9: Corresponde to the uncropped image for figure 4A: ABCA1 in PHT cells (antibody: NB400-105, Novus Biological)

Supplementary figure 10

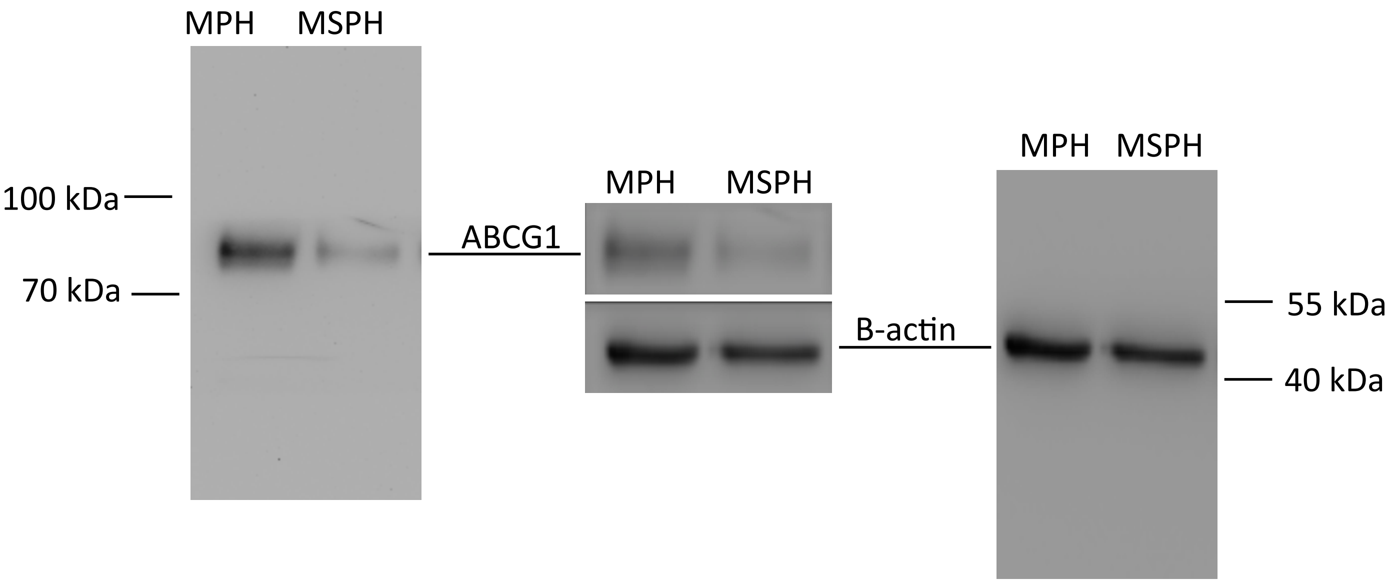

Supplementary figure 10: Corresponde to the uncropped image for figure 4B : ABCG1 in PHT cells (antibody: NB400-132, Novus Biological)

Supplementary figure 11

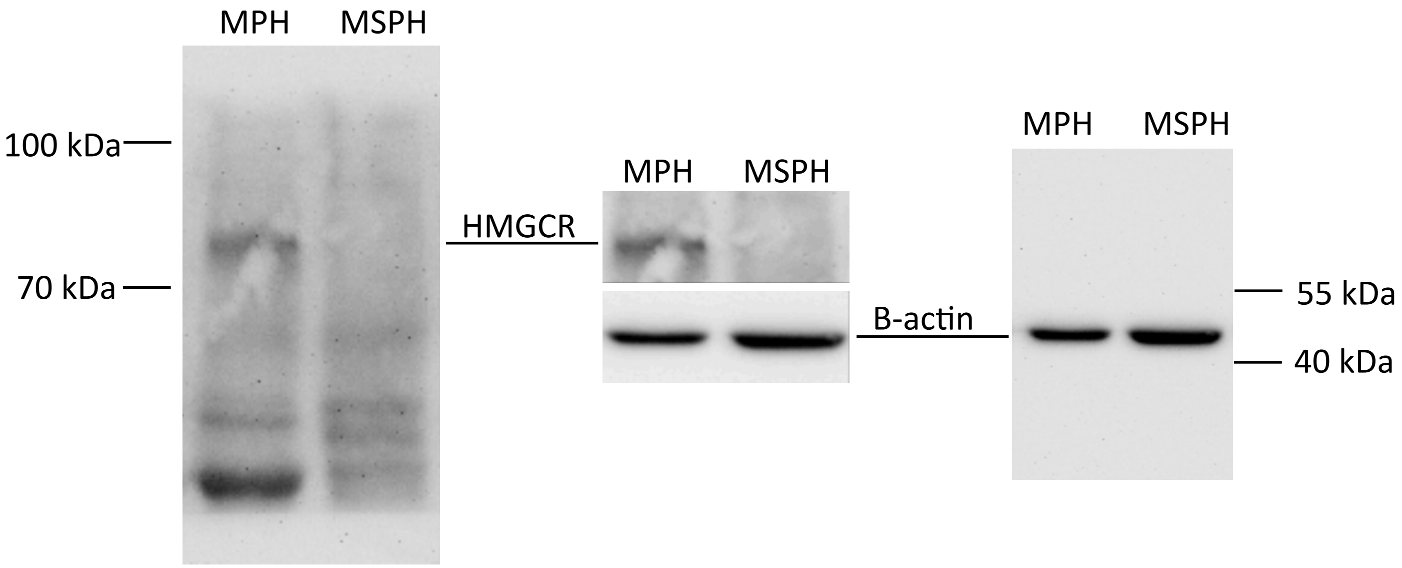

Supplementary figure 11: Corresponde to the uncropped image for figure 5C : HMGCR in PHT cells (antibody: sc271595, Santa Cruz Biotechnology)
